# Potent stimulation of fibroblast growth factor 19 expression in the human ileum by bile acids

**DOI:** 10.1152/ajpgi.00398.2012

**Published:** 2013-03-21

**Authors:** Justine H. Zhang, Jonathan D. Nolan, Sarah L. Kennie, Ian M. Johnston, Tracy Dew, Peter H. Dixon, Catherine Williamson, Julian R. F. Walters

**Affiliations:** ^1^Section of Hepatology and Gastroenterology, Department of Medicine,; ^2^Obstetric Medicine, Imperial College London and Imperial College Healthcare, London;; ^3^Clinical Biochemistry, King's College Hospital, London, United Kingdom

**Keywords:** organ culture, intestine, enterohepatic circulation, bile acid diarrhea

## Abstract

Fibroblast growth factor 19 (FGF19) is proposed to be a negative feedback regulator of hepatic bile acid (BA) synthesis. We aimed to clarify the distribution of FGF19 expression in human intestine and to investigate induction in a novel explant system. Ileal and colonic mucosal biopsies were obtained at endoscopy and analyzed for FGF19 transcript expression. Primary explants were incubated with physiological concentrations of various BA for up to 6 h, and expression of FGF19 and other genes was determined. FGF19 transcripts were detected in ileum but were unquantifiable in colon. No loss of FGF19 mRNA occurred as a consequence of the explant system. Ileal FGF19 transcript expression was induced 350-fold by 50 μM chenodeoxycholate (CDCA, *n* = 24, *P* < 0.0001) and 161-fold by 50 μM glycochenodeoxycholate (GCDCA, *n* = 12, *P* = 0.0005). The responses of other genes to CDCA or GCDCA (50 μM) were smaller: median increases of ileal bile acid binding protein, organic solute transporter-α and -β, and short heterodimer partner were 2.4- to 4.0-fold; apical membrane sodium bile acid transporter and farnesoid X receptor (FXR) showed little change. The EC50 for FGF19 transcript induction by CDCA was 20 μM. FGF19 protein concentrations were significantly higher in the culture fluid from BA-stimulated explants. FGF19 induction with cholate was 81% of that found with CDCA, but deoxycholate (40%) and lithocholate (4%) were significantly less potent. The synthetic FXR agonist obeticholic acid was much more potent than CDCA with a 70-fold FGF19 stimulation at 1 μM. We concluded that FGF19 expression in human ileum is very highly responsive to BA. Changes in FGF19 induction are a potential mechanism involved in disorders of BA homeostasis.

bile acids (BA) undergo an enterohepatic circulation. They are synthesized in the liver, secreted into the duodenum, reabsorbed in the terminal ileum, and recirculated back to the liver via the portal vein. Synthesis of BA is regulated by a negative feedback mechanism (16), involving in part the enteric hormone fibroblast growth factor 19 (FGF19) in humans (35) and its orthologue FGF15 in mice (13). FGF15/FGF19 are produced in response to the absorption of BA (24) and subsequently act on the liver to inhibit cholesterol 7α hydroxylase (CYP7A1) (16, 35), the rate-limiting enzyme in the classical pathway of BA synthesis.

In the mouse, FGF15 expression is abundant in ileum (10), and, except for a small amount of FGF15 expression in jejunum, it is undetectable in other tissues. In humans, FGF19 expression was reported in ileum, but was not reliably detected in duodenum (1). Recently, FGF19 expression has also been reported in human gall bladder and biliary tissues (43).

BA have been shown to stimulate the transcription of FGF15/FGF19 through a nuclear receptor, farnesoid X receptor (FXR, NR1H4) in mouse ileum and cultured human cell lines (13–15). Concentrations of the major physiological primary BA, unconjugated chenodeoxycholic acid (CDCA), ranging from 3–100 μmol/l, have been demonstrated to be effective in activating FXR-mediated BA-response elements in reporter genes in human CV-1 cell lines (25, 31, 40). However, FXR activation was only observed with taurine- and glycine-conjugated BA when the cell lines were also transfected with the apical membrane sodium bile acid transporter (ASBT), which would allow uptake of these more polar conjugated BA. FXR is expressed throughout enterohepatic tissues with clearly defined regulatory roles in BA homeostasis in the liver and the ileum (15).

In humans, serum levels of FGF19 are thought to be dependent on the transintestinal flux of BA, based on the serum peaks observed 90–120 min after the postprandial rise in serum BA. In support of this, serum FGF19 was found to decrease after the treatment with a BA-binding resin and to increase upon oral administration of CDCA (24).

Enterocytes in the terminal ileum are specialized to take up conjugated BA such as glyco-chenodeoxycholic acid (GCDCA) and tauro-chenodeoxycholic acid from the intestinal lumen. This function is mediated by the expression of the ileal ASBT. BA then bind to the ileal bile acid-binding protein (IBABP) and are extruded at the basolateral membrane by the organic solute transporter (OST)-α/β heterodimer (8).

Reduced fasting levels of FGF19 have been observed in clinical situations of BA malabsorption (39). This has been shown in patients who have undergone previous ileal resection or ileal Crohn's disease (20). However, lower FGF19 levels are also observed in patients with the condition known as primary (idiopathic) BA diarrhea (or malabsorption), which. despite its name, is not clearly associated with any malabsorptive defect in most patients (36). We have proposed an alternative mechanism for the diarrhea in this condition. Impaired FGF19 feedback inhibition causes excessive BA synthesis, which exceeds the normal capacity for ileal reabsorption, and so proceeds to induce diarrhea in the colon (39). This is consistent with the observation that the BA pool size is increased in these patients (37).

This study aimed to confirm the expression of FGF19 transcripts in the human ileum and to investigate levels of FGF19 expression in other parts of the human intestine (10). Using short-term tissue cultures of intestinal biopsies obtained during ileo-colonoscopy, we hypothesized that FGF19 expression would be induced with CDCA and its conjugate GCDCA (31, 32) and proposed to compare this with the other natural primary BA, cholic acid, and the secondary BAs, deoxycholic acid (DCA) and lithocholic acid (LCA), which differ as FXR agonists. We also included study of the potent synthetic FXR agonist obeticholic acid (OCA) (23, 33). Research into the BA-mediated induction of ileal FGF19 may further elucidate the pathophysiology of gastrointestinal diseases associated with ileal dysfunction, such as Crohn's disease and primary BA diarrhea, which have previously been associated with reduced circulating levels of serum FGF19 (19, 39).

## MATERIALS AND METHODS

### 

#### Subjects.

In total, 42 subjects were recruited from patients undergoing routine ileo-colonoscopy. Subjects gave informed, written consent. All subjects had fasted overnight in preparation for colonoscopy and had received bowel preparation, usually with oral Senna and magnesium citrate. Exclusion criteria were as follows: patients under 18 yr old, patients on anticoagulant or dual-antiplatelet therapy, and patients with microscopic or macroscopic evidence of ileal pathology, as shown on histological examination. Intestinal mucosal biopsies were taken with the same type of disposable biopsy forceps (radial jaw 3; Boston Scientific, Hemel Hempstead, UK), giving standard-sized mucosal samples. The study was approved by the local Institutional Review Board, the Hammersmith, Queen Charlotte's, and Chelsea Hospitals' Research Ethics Committee.

#### FGF19 baseline expression studies.

For FGF19 baseline expression studies, three terminal ileum biopsies were obtained from 11 patients. Additionally, in each patient, 3–6 further biopsies were obtained from up to two of the following colonic sites: cecum, ascending colon, transverse colon, or sigmoid colon. These were stored in RNAlater (Sigma-Aldrich, Gillingham, UK) at −80°C before RNA extraction.

#### Ileal explant studies.

We recruited 28 patients for ileal explant studies, one of whom was also a patient in the FGF19 baseline expression studies. In each patient, up to 10 biopsies were obtained from the terminal ileum for routine histology and research studies. Biopsies were placed in preoxygenated Dulbecco modified Eagle's medium (PAA Laboratories, Somerset, UK) and transferred to the laboratory within 5 min, on ice. Ileal explants were placed in groups of two to three on tissue culture inserts (MilliCell HA Culture plate inserts, Sigma-Aldrich) in six-well culture plates (Nunclon Δ Surface multidishes; VWR International, Lutterworth, UK). Explants were sustained in 2 ml of culture media (Dulbecco modified Eagle's medium), 10% heat-inactivated fetal calf serum, 100 units/ml penicillin, 100 μg/ml streptomycin, 50 μg/ml leupeptin, 50 μg/ml soybean trypsin inhibitor, and 1 mM phenylmethylsulfonyl fluoride, incubated at 37°C, and gassed with 95% O_2_-5% CO_2_ at 0 h and every hour thereafter. At the end of the experiment, biopsies were placed in RNAlater, stored at 4°C overnight, and then transferred the next day to storage at −80°C before RNA extraction. Conditioned media were stored at −80°C before analysis of FGF19 protein content by specific ELISA. This experimental setup is similar to a previous successful setup described by Balesaria et al. (2) for human duodenal transcriptional responses to stimulation by vitamin D.

In each experiment, one group of biopsies was incubated in complete media without the addition of exogenous BA (control group). Subsequent groups of biopsies were incubated with added BA, in the following combinations: 50 μM CDCA in parallel with 50 μM GCDCA for 6 h; 1–100 μM varying concentrations of CDCA or GCDCA for 6 h; 50 μM CDCA or GCDCA for varying incubation times between 1 and 6 h; 50 μM of other physiological BA, including CA, DCA, and LCA, and the semisynthetic bile acid FXR agonist, 1 or 20 μM OCA. These concentrations were chosen because they are physiological and reflect concentrations at which CDCA has been shown to be an agonist for FXR (31).

#### RNA extraction.

RNA extraction was performed using a RNeasy Mini kit (Qiagen, Crawley, UK) on biopsies from the FGF19 expression studies and ileal explant studies. Extraction was carried out according to the manufacturer's protocol. To minimize genomic DNA contamination, further RNA purification was performed on column using a RNase-free DNase set (Qiagen). RNA quantification and assessment of RNA purity was performed using a ThermoScientific NanoDrop 1000 Spectrophotometer.

#### cDNA synthesis.

Synthesis of cDNA from RNA was carried out using a SuperScript kit (Invitrogen, Paisley, UK). Reaction volumes of 20 μl were used, containing 1 μg RNA, 1 μl dNTP mix (10 mM), 1 μl random hexamers (50 ng/μl), 2 μl 10× RT buffer, 4 μl MgCl_2_ (25 mM), 2 μl DTT (0.1 M), 1 μl RNaseOUT (40 U/μl), 1 μl SuperScript II RT (50 U/μl), and diethylpyrocarbonate (DEPC)-treated water. Reverse transcription was performed at 42°C for 50 min according to the manufacturer's instructions. Minus reverse-transcriptase controls were included, where SuperScript II RT was replaced with 1 μl DEPC-treated water. Resultant cDNA was treated with 1 μl *Escherichia coli* RNase H (2 U/μl) for 20 min at 37°C. Final cDNA was diluted twofold before proceeding with RT-PCR.

#### Quantitative real-time RT-PCR.

Amplification of cDNA was performed using SYBR Green JumpStart Taq Ready Mix (Sigma-Aldrich), working primer stock concentrations of 10 μM, and cDNA volumes of 1 μl per well, in an Applied Biosystems 7900HT Fast cycler (Warrington, UK). Primer sequences and characteristics are shown in Table 1. Singleplex RT-PCR assays were performed in triplicate in 25-μl reaction volumes, using MicroAmp Fast Optical 96-well reaction plates (Applied Biosystems, UK) or in 8 μl reaction volumes using MicroAmp Optical 384-Well reaction plates (Applied Biosystems). Each plate was run with minus reverse-transcriptase controls and PCR controls (1 μl DEPC-treated water added instead of 1 μl cDNA). The thermal cycling program was as follows: 10 min of 95°C (to activate Taq polymerase), then 40 cycles of 15 s at 95°C, 1 min at 60°C, and 1 min at 72°C, ramping at 1.4°C/s between each step of the cycle. Triplicates where differences in cycle time (Ct) values exceed 0.5 Ct were analyzed for outliers, and outliers were removed from calculations. Results were analyzed using the comparative Ct method (also known as the ΔΔCt method) to give fold changes of FGF19 expression relative to the control sample in each patient. GAPDH was used as the endogenous control in each sample to normalize expression.

**Table 1.** Primer sequences used in quantitative real-time RT-PCR

Primer	Sequence (5′-3′)
Human FGF19, forward	CGG TAC CTC TGC ATG GGC
Human FGF19, reverse	CCA TCT GGG CGG ATC TCC
Human GAPDH, forward	CGA CCA CTT TGT CAA GCT CA
Human GAPDH, reverse	AGG GGA GAT TCA GTG TGG TG
Human ASBT, forward	GCC CCA AAA AGC AAA GAT CA
Human ASBT, reverse	GCT ATG AGC ACA ATG AGG ATG
Human IBABP, forward	TCA GAG ATC GTG GGT GAC AA
Human IBABP, reverse	TCA CGC GCT CAT AGG TCA
Human SHP, forward	AGG GAC CAT CCT CTT CAA CC
Human SHP, reverse	TTC ACA CAG CAC CCA GTG AG
Human FXR, forward	AGG ATT TCA GAC TTT GGA CCA TGA
Human FXR, reverse	TGC CCA GAC GGA AGT TTC TTA TT
Human OST-α, forward	AGA TTG CTT GTT CGC CTC C
Human OST-α, reverse	ATT CGT GTC AGC ACA GTC ATT
Human OST-β, forward	CAG GAG CTG CTG GAA GAG AT
Human OST-β, reverse	GAC CAT GCT TAT AAT GAC CAC

FGF, fibroblast growth factor; ASBT, apical membrane sodium bile acid transporter; IBABP, ileal bile acid-binding protein; SHP, short heterodimer partner; FXR, farnesoid X receptor; OST, organic solute transporter.

#### ELISA.

ELISA was used to measure FGF19 protein concentration in supernatant (FGF19 Quantikine ELISA kit; R&D Systems, Minneapolis, MN), according to the manufacturer's protocols. FGF19 protein values were normalized to the number of biopsies (2–3) incubated in each culture well.

#### Statistical analyses.

Statistical analyses were performed in GraphPad Prism Version 5.04 software (GraphPad Software, San Diego, CA). Nonparametric two-tailed tests (Wilcoxon and Mann-Whitney tests) were used as appropriate. *P* values <0.05 were considered significant. Correlation of results with clinical variables was tested by calculating Spearman's rank correlation coefficient.

## RESULTS

### 

#### FGF19 is expressed in human ileum but is undetectable in human colon.

We first determined FGF19 expression in the human intestine. Of the 11 ileal samples, all expressed FGF19, with a mean Ct of 29.9, showing lower expression than GAPDH (mean Ct 22.5). Standard error of ΔCt values for FGF19 from ileal samples was 1.141, reflecting that the samples had a low variability of up to twofold difference in FGF19 expression. Inactive Crohn's disease was present in two patients, but neither of these patients had significantly different ileal FGF19 expression levels from the nine patients without Crohn's disease. In all the cecal, ascending, transverse, and sigmoid colon samples, FGF19 was either completely undetectable, or expression was at the limits of reliable quantification (Table 2). Expression of FGF19 was less than 0.004 of that seen in the corresponding ileum.

**Table 2.** Cycle threshold (Ct) values at different biopsy sites

Biopsy Site		*n*	Median Ct
Ileum		8	29.9
Colon	Ascending	4	>40
	Cecum	1	>40
	Transverse	1	>40
	Sigmoid	6	37.7

Ct values in the gastrointestinal tract show the cycle at which FGF19 amplification occurred (the higher the Ct, the later the amplification, so the lower the gene expression). Ct values >40 cycles signify undetectable gene expression. Ct values >35 cycles are widely considered to signify gene expression at the limits of reliable quantification. *n* = number of subjects.

#### Six-hour incubation does not result in loss of FGF19 mRNA.

**Fig. 1.**
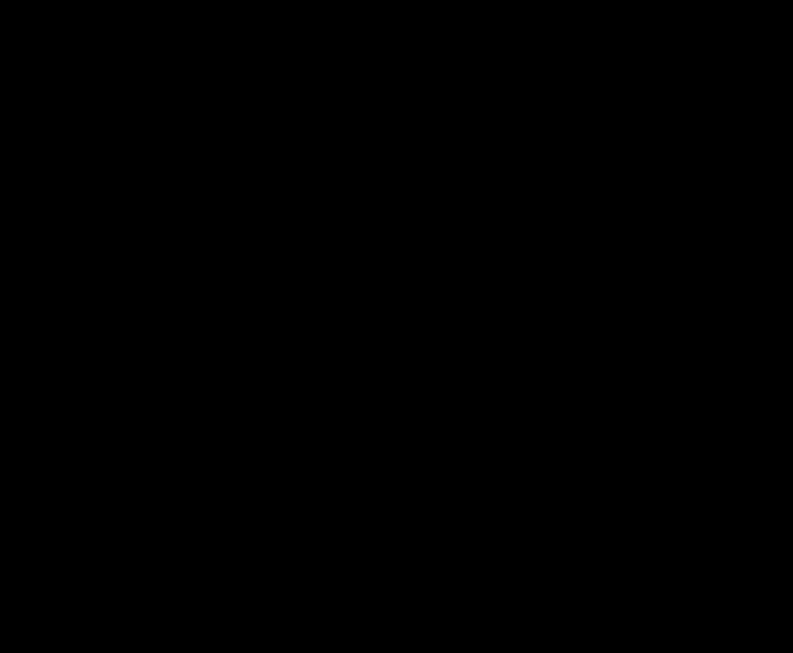
Fibroblast growth factor 19 (FGF19) mRNA expression is unchanged during 6-h explant culture. *Left*: FGF19 mRNA in ileal biopsies stored for immediate processing (basal expression, *n* = 11), and those incubated in control culture media for 6 h (control incubation, *n* = 35) are shown, together with medians and interquartile ranges. There is no statistically significant difference between them, *P* = 0.5, Mann-Whitney test. *Right*: relative quantities of FGF19 mRNA are similar in paired samples taken from the same patient. Basal expression and that after control incubation are shown (*n* = 4). The quantity of FGF19 mRNA relative to GAPDH has been calculated using the equation 2^−(ΔCt)^. AU, arbitrary units.

Groups of ileal samples were incubated for 6 h in the explant system, with either 50 μM CDCA, 50 μM GCDCA, or no BA (unstimulated controls). Unstimulated ileum did not show a difference in FGF19 expression after a 6-h incubation compared with other ileal samples that had not undergone incubation (*P* = 0.26, Mann-Whitney test) (Fig. 1, *left*). We confirmed this in four patients with matched samples that were either immediately processed or incubated for 6 h (Fig. 1, *right*). This shows that incubation of ileum does not result in loss of FGF19 mRNA, providing support for the robustness of this experimental setup.

#### FGF19 transcript expression in human ileum is strongly induced by CDCA and GCDCA.

**Fig. 2.**
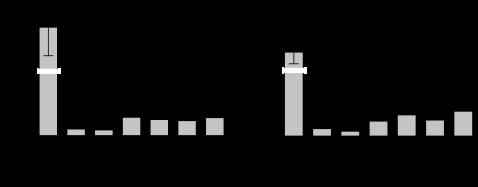
*A*: transcript expression after 6-h incubation with 50 μM chenodeoxycholate (CDCA). Number of subjects (*n*) and *P* values were: FGF19 (*n* = 23, *P* < 0.0001); apical membrane sodium bile acid transporter (ASBT) (*n* = 4, *P* = 0.23); farnesoid X receptor (FXR) (*n* = 4, *P* = 0.30); ileal bile acid-binding protein (IBABP) (*n* = 4, *P* = 0.02); organic solute transporter (OST)-α (*n* = 3, *P* = 0.11); OST-β (*n* = 4, *P* = 0.02); short heterodimer partner (SHP) (*n* = 4, *P* = 0.03). *B*: transcript expression after 6-h incubation with 50 μM glycochenodeoxycholate (GCDCA). *n* and *P* values: FGF19 (*n* = 12, *P* = 0.0005); ASBT (*n* = 4, *P* = 0.40); FXR (*n* = 4, *P* = 0.52); IBABP (*n* = 4, *P* = 0.06); OST-α (*n* = 3, *P* = 0.10); OST-β (*n* = 4, *P* = 0.07); SHP (*n* = 4, *P* = 0.06). Data are expressed as median and interquartile ranges of expression ratio fold changes, calibrated to matched unstimulated control samples, relative to GAPDH. Control sample mean Ct values for GAPDH, FGF19, ASBT, FXR, IBABP, OST-α, OST-β, and SHP were 22.5, 29.5, 21.9, 24.9, 20.7, 25.7, 23.9, and 27.8, respectively. *P* values were calculated from ΔCT values by paired *t*-tests.

After 6-h incubation, FGF19 expression increased significantly, with a median induction of 350-fold by 50 μM CDCA (*n* = 24, *P* < 0.0001, two-tailed Wilcoxon test), and 161-fold by 50 μM GCDCA (*n* = 12, *P* = 0.0005). There was no significant difference in induction of FGF19 expression between CDCA and GCDCA-stimulated ileum (*P* = 0.3, two-tailed Wilcoxon test). Results are expressed graphically in Fig. 2.

The responses of other relevant genes to CDCA and GCDCA at 50 μM (*n* = 3 - 4) showed median increases for IBABP, OST-α, OST-β and short heterodimer partner (SHP) between 2.4- and 4.0-fold, whereas ASBT and FXR showed little change (Fig. 2). The unstimulated expression of FGF19 was lower (with a high Ct value) than the other genes, but two other FXR-responsive genes differed greatly, with SHP similar to FGF19, but IBABP much higher (with a lower Ct value). Responses to CDCA and GCDCA at 100 μM appeared similar to those at 50 μM (data not shown).

**Fig. 3.**
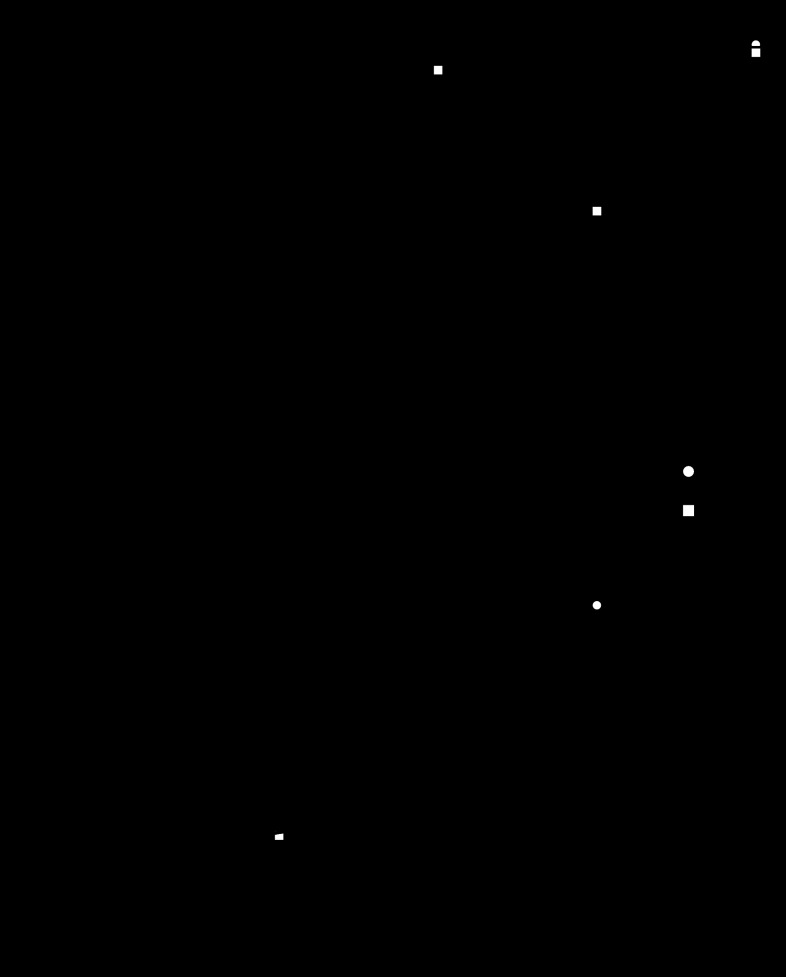
Dose-ranging studies of CDCA induction of FGF19 mRNA. Graph shows pooled data from 10 explant studies that were incubated with 2–5 different concentrations of CDCA in parallel. Concentrations ranged from 1–100 μM. Data points are shown from each individual explant from each individual patient (see data points *A*–*J*). Fold changes in mRNA were converted to a percentage of maximum response, which was observed at 50 μM in those studies whose data points are shown in the oval ring and 100 μM for those shown in the rectangular box.

Incubations with a range of two to five different CDCA concentrations (5–100 μM) were performed in parallel in 10 patients. The induction of FGF19 mRNA was similar at 100 μM and 50 μM CDCA (Fig. 3). A nonlinear, least squares, sigmoidal curve-fitting program was used (GraphPad Prism 5) to derive the EC50 for studies that included four or five different CDCA concentrations (*n* = 4). The median EC50 was 20 μM (range 16–52).

**Fig. 4.**
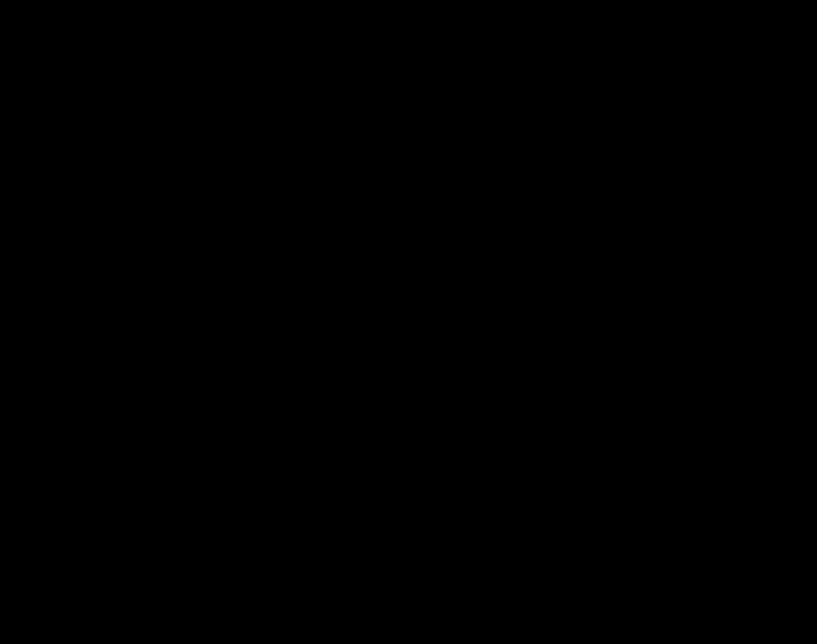
Time courses (*n* = 4) showing FGF19 expression increased over the course of 6-h incubation with 50 μM CDCA. Incubations with CDCA were for 0.5 h, 1 h, 1.5 h, 2 h, 3 h, or 6 h. Explants were placed into RNAlater immediately after incubation. FGF19 expression at 0.5 h and 1 h showed no significant change, at 1.5 h and 2 h was <10-fold, at 3 h was >40-fold, and at 6 h was dramatically higher at several hundred fold. Data are expressed as median fold changes, calibrated to matched unstimulated control samples, relative to GAPDH. Interquartile ranges are shown where appropriate.

Time-course experiments showed that FGF19 expression increased over the course of 6-h incubation with 50 μM CDCA (Fig. 4). Stimulation of FGF19 expression increased from under 10-fold at 2 h to median >40-fold at 3 h, to several hundred fold at 6 h incubation.

#### Ex vivo FGF19 protein release from ileal explants.

**Fig. 5.**
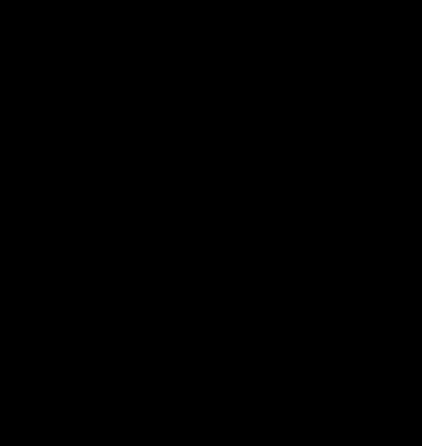
Increases in FGF19 protein in culture fluid after bile acid (BA) incubations. *A*: CDCA (*n* = 10), GCDCA (*n* = 9). FGF19 protein levels were measured by specific ELISA (FGF19 Quantikine ELISA kit; R&D Systems, Minneapolis, MN) at the end of 6-h incubations. FGF19 protein levels (pg/explant) are higher in the culture fluid from every BA-stimulated explant compared with their respective paired control explant. *B*: correlation between the culture fluid protein levels and fold changes in mRNA levels of FGF19 CDCA (*n* = 10) and GCDCA (*n* = 9).

Concentrations of FGF19 protein were measured from culture fluid in 16 of the explant studies. After 6-h incubation, the median level of FGF19 protein was significantly higher (*n* = 10, *P* = 0.003, two-tailed Mann-Whitney) in the culture fluid from explants incubated with 50 μM CDCA (56 pg/explant, range 8–169) compared with the control explants (7 pg/explant, range 0–46). The median level of FGF19 was also significantly higher (*n* = 9, *P* = 0.002, two-tailed Mann-Whitney) in the culture fluid from explants incubated with 50 μM of GCDCA (median 24 pg/explant, range 3–113) compared with the matched control explants (6 pg/explant, range 0–13), (Fig. 5*A*). There was also a significant correlation between the culture fluid protein and fold changes in mRNA of FGF19 (CDCA: *n* = 10, *r* = 0.70, *P* = 0.0005; GCDCA: *n* = 9, *r* = 0.76, *P* = 0.0003, Spearman's rank test; Fig. 5*B*).

#### The induction of FGF19 mRNA in human ileum by different BA correlates with their relative potencies as FXR agonists.

**Fig. 6.**
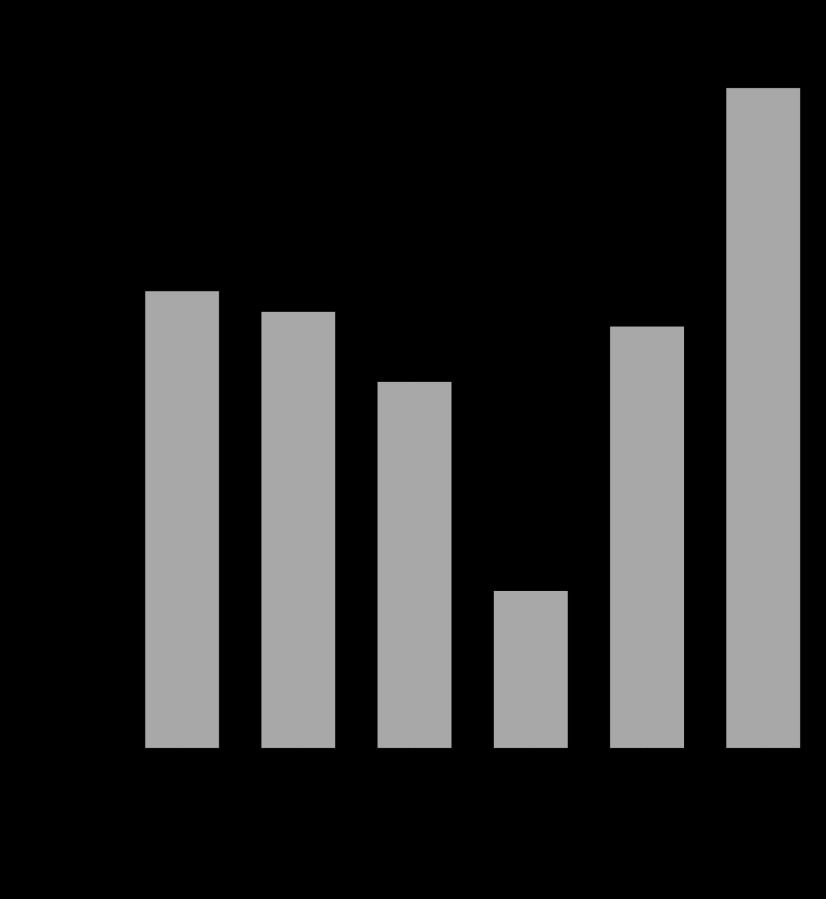
Relative potencies of different BAs at inducing FGF19 mRNA expression. Relative induction of FGF19 mRNA is expressed as a percentage of the induction observed in paired explants stimulated with 50 μM CDCA. Mean percentages and range are shown. The induction of FGF19 mRNA compared with paired CDCA stimulated explants was 81% with cholic acid (CA) (*n* = 4), 40% with deoxycholic acid (DCA) (*n* = 3) and 4% with lithocholic acid (LCA) (*n* = 5). Statistical significance: CA, *P* = 0.5; DCA, *P* = 0.03; LCA, *P* < 0.001; paired *t*-tests % vs. CDCA 50 μM. With obeticholic acid (OCA), induction of FGF19 at OCA 1 μM was comparable with that found with CDCA 50 μM (*n* = 3), and induction with OCA 20 μM was much greater than that found with CDCA 20 μM or 50 μM (*n* = 3).

Some ileal explants were incubated with CDCA in parallel with other natural BA (CA, DCA, and LCA) or the synthetic FXR agonist OCA. CDCA and CA were found to have a broadly similar potency at inducing the expression of FGF19 mRNA. The mean induction of FGF19 mRNA expression in explants incubated with 50 μM CA was 81% of that seen with respective paired CDCA incubations, whereas significantly lower induction was found with DCA (40%) and with LCA at 4% (Fig. 6).

After incubations with 1 μM OCA for 6 h, a median fold change of 70 (range 40–258, *n* = 3) was observed although no induction in FGF19 mRNA expression was observed with 1 μM CDCA (*n* = 3). In paired incubations involving both 20 μM CDCA and 20 μM OCA, median induction in FGF19 mRNA expression was five times greater with OCA (range 3–45, *n* = 3). The greater potency of OCA compared with CDCA, apparent at the lower dose of 1 μM, indicates that OCA has a much lower EC50.

#### Induction of FGF19 expression by BA displays interperson variability.

There was variability in different subjects in the magnitude of ileal FGF19 induction after BA stimulation (CDCA-stimulated samples: range 48–939; GCDCA-stimulated samples: range 56–1,065). This may represent differences in equilibrium levels for BA homeostasis between individuals. However, no significant associations between the magnitude of FGF19 induction with age, sex, or stool frequency (≥3/day vs. <3/day) were observed.

## DISCUSSION

This study has confirmed human ileal expression of FGF19 and has shown for the first time that ileal FGF19 is greatly stimulated by physiological concentrations of BA. Before this study, the effect of BA on FGF19 transcription in human ileum was unknown because no system existed to investigate this. We have now developed an explant system that is functional and does not result in loss of FGF19 mRNA. The potential applications of this system are numerous, as it enables investigation of FGF19 stimulation in human ileum under controlled conditions. This system is more akin to natural human physiological conditions compared with previous systems that used mouse ileum, hepatocytes, or human cell lines. The use of explanted human tissue in this system also allows ileal FGF19 stimulation to be directly linked to subjects, so that ileal FGF19 stimulation may be investigated in different disease states.

We have demonstrated that stimulation of the human ileum with 50 μM CDCA or 50 μM GCDCA induces FGF19 transcript expression in all individuals, with a median induction of ∼300-fold. This is greater than the ∼80-fold FGF15 induction previously shown in mouse ileum after stimulation with a synthetic FXR agonist GW4064 (15). Species differences and experimental design may have contributed to the magnitude difference.

The large induction in ileal FGF19 expression we have shown is also larger than that previously observed in primary cultures of human hepatocytes (35). In those experiments, control samples had low expression of FGF19, but a dose-dependent increase in FGF19 mRNA levels was seen, of about 80–1,200-fold after 6–24-h incubation with CDCA (50 μM) or with GW4064 (1 μM).

We also found that FGF19 is expressed only at low levels in human colon, at the limits of reliable quantification, suggesting that the colon is unlikely to be important in mediating this intestine-liver hormonal signaling for the maintenance of BA homeostasis. FXR, the bile acid-activated nuclear receptor, is expressed in more locations in the gastrointestinal tract than FGF19, including expression in the colon (6). This discrepancy between FGF19 and FXR expression indicates that other factors must also be involved in the transcriptional control of FGF19, to produce high levels in ileum. Other transcription factors that may be candidates for regulating the distribution of FGF19 expression include homeobox transcription factors and GATA4 (3–5, 7, 38).

An FXR-responsive element was first described in the second intron of the *FGF19* gene and mediated reporter gene activation in HuH7 cells (13). This is conserved in the mouse *Fgf15* gene. More recently, three novel FXR-responsive elements have been described in reporter and binding assays in the 5′-flanking region of the FGF19 gene, which are not conserved in the mouse (28). These binding assays used recombinant FXR proteins and specific FXR antibodies, and the FXR response elements activate reporter gene expression when stimulated with the FXR agonists, CDCA or GW4064, to a much greater extent than LCA. LCA alone has a weak effect on a possible pregnane X receptor (PXR) element.

The large 300-fold changes in FGF19 mRNA expression appear to dwarf the <4-fold changes of the other BA-regulated genes investigated in this study. IBABP, OST-α, and OST-β genes have been shown to have FXR-mediated transcriptional responses, which are predicted to lead to an increase BA-ileal fluxes, but their induction in our system was only a fraction of FGF19. SHP, involved in BA-mediated transcription regulation, and previously shown to be highly induced in mouse ileum (15), was also stimulated much less than FGF19. These differences in magnitude suggest that FGF19 may have the dominant role in the negative feedback regulation of the BA enterohepatic circulation, supporting the mouse data on FGF15 (14). ASBT expression has previously been shown to be negatively regulated by BA indirectly through SHP in humans (30), and FGF15/19 in mice and human cell lines can also repress ASBT promoter activation, RNA, and protein expression (34).

One of the main effects of FGF19 is the negative feedback regulation of BA synthesis, and, for this to be achieved, FGF19 transcripts must be translated into protein and released into the portal circulation. We have attempted to look at this by quantifying protein in the culture fluid. Our results reveal significant differences in magnitude between the ≈300-fold change in transcript expression and the much lower ≈8-fold-changes in FGF19 protein. This is could be due to the extra time needed for translation and export of the protein but may reflect some additional regulation of those processes including transcript stabilization, posttranslational modification, secretion, and stability of the protein in the culture fluid.

It is also important to note that the measurements we have made in our explant system are steady-state transcript levels at a particular time, rather than direct measures of FGF19 synthesis or degradation. To get a better idea of the dynamics of FGF19 transcript expression, we conducted limited time course experiments, which showed that FGF19 transcript expressions were not increased by 1 h of incubation but, after then, continued to increase up to 6 h. This time course is typical for transcription and shows, in this experimental situation, that, as the length of time of BA exposure increases, more FGF19 transcripts are synthesized than degraded, resulting in a net increase in FGF19 transcript levels.

We studied both unconjugated CDCA and conjugated GCDCA in most experiments because of concerns that GCDCA would only be taken up in cells that expressed ASBT, whereas CDCA could permeate the cell membrane without specific transport systems (25, 31, 40). Expression of ASBT is a key finding in the ileum with its uptake and transcellular flux of BA but is known to be reduced in disease states (17). In our experiments, stimulation was found in ileal explants in all cases with GCDCA, but we have not included any subjects with identified ileal disease.

It is widely acknowledged that the most potent physiological FXR agonist is CDCA, but there has been some ambiguity regarding the relative potency of CA as an FXR agonist (21, 25, 31, 40). Minimal FXR activation was found in nonintestinal CV-1 cells with CA, compared with CDCA and secondary BA (31), and similar findings were reported using human FXR and a reporter gene in HepG2 cells (25). However, other groups have shown that CA is able to stimulate FXR responses (21, 40). Here we have shown that FGF19 is strongly induced with both CDCA and CA and, to a lesser extent, with DCA. Our study used human tissue in a physiological system where BA transport occurs, giving confidence that this system generated accurate data regarding human ileum. Nonetheless, putting this data into the context of previous findings does raise the question of whether any alternative mechanisms to FXR activation may be responsible for inducing FGF19.

LCA was shown to be poor at inducing FGF19 expression in our human ileal explants. Our data are in keeping with the reports that LCA binds to, and antagonizes activation of, FXR (42) but does not concur with some other findings (31). A more recent study reported that LCA might induce FGF19 expression independently of FXR following PXR (NR1I2) activation (41). However, LCA has toxic properties (42), which could have had a negative bearing on the stability of our ileal explant system. Further data are required to fully elucidate the roles of secondary BA in inducing FGF19. Part of their action is to antagonize FXR activation by other BA. Certain other FXR antagonists have been used in experiments with cell lines and in mice, and their use in this system should be considered. Whether their uptake would be sufficient in the short time frame available and whether preincubation would be needed is not known.

A number of studies indicate a possible candidate for FXR-independent induction of FGF19 if a different selectivity had been shown for various BA. The G protein-coupled membrane receptor, BG37/TGR5, has been found to be expressed in ileum and other tissues, binds certain BA, and increases intracellular cyclic adenosine monophosphate (26). No link has been shown between TGR5 activation and downstream FGF15/19 expression or for any other FXR target genes. Interestingly, one study (22) did report a possible “crosstalk” scenario between FGF15 and TGR5 on gallbladder motility. Intestinal TGR5 appears to be linked to glucose and lipid homeostasis (18) rather than BA regulation.

This study successfully confirmed the implications of previous reports (27, 32) showing OCA to be a potent ligand for FXR. OCA strongly induced FGF19 in all subjects at both of the studied concentrations. This supports the potential therapeutic role of OCA as a novel FXR agonist in gastrointestinal disease with defective FXR activation/FGF19 induction patterns. OCA enters the enterohepatic circulation (12) and thus can be used to regulate hepatic BA synthesis. Previous reports have focused on cholestatic diseases (21) and, more recently, metabolic disorders (9, 29), but we would also anticipate a role for OCA in gastrointestinal disorders including primary BA diarrhea.

The individual variability in ileal FGF19 induction by both CDCA and GCDCA indicates different levels of BA homeostatic equilibrium (or disequilibrium). Some people may thus be more susceptible to disorders of BA homeostasis than others. Primary BA diarrhea is likely to be a disease state that is more common in individuals who have disequilibrium of BA homeostasis. Our group has demonstrated that patients with primary BA diarrhea had lower median fasting serum FGF19 levels than controls and proposed that primary BA diarrhea may be due to inadequate FGF19 expression, resulting in impaired negative feedback and consequent overflow of BA into the colon (39). One previous study demonstrated a significant depletion in CA levels in patients with primary BA diarrhea compared with controls (11). This could have been important if our findings had suggested a higher potency for CA in inducing FGF19 compared with CDCA but was not the case, as CDCA and CA were not significantly different. We did not recruit any patients with diagnosed primary BA diarrhea for this study but will be investigating its pathogenesis in further studies, looking at basal and stimulated FGF19 secretion. Studies in patients with ileal inflammatory disease or resection, as in Crohn's, and perhaps those with other disorders of bile acid regulation, such as gallstone disease, may also be of interest.

In summary, this study has found that FGF19 is highly expressed in human ileum and increases more than 300-fold in 6 h after stimulation with the potent FXR-activating BA, CDCA, and OCA. We have achieved this through an explant system, which in future can be used to investigate the role of FGF19 in disorders of BA homeostasis.

## GRANTS

I. Johnston was funded by the Bardhan Research & Education Trust and J. Nolan by the Broad Medical Research Program. We also acknowledge support from the NIHR Biomedical Research Centre funding scheme and the NIHR/Wellcome Trust Clinical Research Facility at Imperial College Healthcare NHS Trust.

## DISCLOSURES

No conflicts of interest, financial or otherwise, are declared by the authors.

## AUTHOR CONTRIBUTIONS

Author contributions: J.H.Z., J.D.N., S.L.K., I.M.J., and J.R.W. conception and design of research; J.H.Z., J.D.N., S.L.K., T.D., and P.H.D. performed experiments; J.H.Z., J.D.N., S.L.K., I.M.J., T.D., P.H.D., C.W., and J.R.W. analyzed data; J.H.Z., J.D.N., S.L.K., I.M.J., P.H.D., C.W., and J.R.W. interpreted results of experiments; J.H.Z., J.D.N., and S.L.K. prepared figures; J.H.Z., J.D.N., S.L.K., and J.R.W. drafted manuscript; J.H.Z., J.D.N., S.L.K., I.M.J., T.D., P.H.D., C.W., and J.R.W. edited and revised manuscript; J.H.Z., J.D.N., S.L.K., I.M.J., T.D., P.H.D., C.W., and J.R.W. approved final version of manuscript.

## References

[1] Balesaria S Pell RJ Abbott LJ Tasleem A Chavele KM Barley NF Khair U Simon A Moriarty KJ Brydon WG Walters JR Exploring possible mechanisms for primary bile acid malabsorption: evidence for different regulation of ileal bile acid transporter transcripts in chronic diarrhoea. Eur J Gastroenterol Hepatol 20: 413–422, 2008 18403943 10.1097/MEG.0b013e3282f41b82

[2] Balesaria S Sangha S Walters JR Human duodenum responses to vitamin D metabolites of TRPV6 and other genes involved in calcium absorption. Am J Physiol Gastrointest Liver Physiol 297: G1193–G1197, 2009 19779013 10.1152/ajpgi.00237.2009 PMC2850091

[3] Barley NF Taylor V Shaw-Smith CJ Chakravarty P Howard A Legon S Walters JR Human ileal bile acid-binding protein promoter and the effects of CDX2. Biochim Biophys Acta 1630: 138–143, 2003 14654244 10.1016/j.bbaexp.2003.09.008

[4] Battle MA Bondow BJ Iverson MA Adams SJ Jandacek RJ Tso P Duncan SA GATA4 is essential for jejunal function in mice. Gastroenterology 135: 1676–1686; e1671, 2008 10.1053/j.gastro.2008.07.074 PMC2844802 18812176

[5] Beuling E Kerkhof IM Nicksa GA Giuffrida MJ Haywood J aan de Kerk DJ Piaseckyj CM Pu WT Buchmiller TL Dawson PA Krasinski SD Conditional Gata4 deletion in mice induces bile acid absorption in the proximal small intestine. Gut 59: 888–895, 2010 20581237 10.1136/gut.2009.204990 PMC2981798

[6] Bookout AL Jeong Y Downes M Yu RT Evans RM Mangelsdorf DJ Anatomical profiling of nuclear receptor expression reveals a hierarchical transcriptional network. Cell 126: 789–799, 2006 16923397 10.1016/j.cell.2006.06.049 PMC6211849

[7] Bosse T Piaseckyj CM Burghard E Fialkovich JJ Rajagopal S Pu WT Krasinski SD Gata4 is essential for the maintenance of jejunal-ileal identities in the adult mouse small intestine. Mol Cell Biol 26: 9060–9070, 2006 16940177 10.1128/MCB.00124-06 PMC1636804

[8] Dawson PA Lan T Rao A Bile acid transporters. J Lipid Res 50: 2340–2357, 2009 19498215 10.1194/jlr.R900012-JLR200 PMC2781307

[9] Fiorucci S Cipriani S Mencarelli A Baldelli F Bifulco G Zampella A Farnesoid X receptor agonist for the treatment of liver and metabolic disorders: focus on 6-ethyl-CDCA. Mini Rev Med Chem 11: 753–762, 2011 21707532 10.2174/138955711796355258

[10] Fon Tacer K Bookout A Ding X Kurosu H John G Wang L Goetz R Mohammadi M Kuro-o M Mangelsdorf D Kliewer S Research resource: comprehensive expression atlas of the fibroblast growth factor system in adult mouse. Mol Endocrinol 24: 2050–2064, 2010 20667984 10.1210/me.2010-0142 PMC2954642

[11] Fracchia M Pellegrino S Secreto P Pera A Galatola G Biliary lipid composition in idiopathic bile acid malabsorption. Gut 43: 812–816, 1998 9824609 10.1136/gut.43.6.812 PMC1727337

[12] Gadaleta RM van Erpecum KJ Oldenburg B Willemsen EC Renooij W Murzilli S Klomp LW Siersema PD Schipper ME Danese S Penna G Laverny G Adorini L Moschetta A van Mil SW Farnesoid X receptor activation inhibits inflammation and preserves the intestinal barrier in inflammatory bowel disease. Gut 60: 463–472, 2011 21242261 10.1136/gut.2010.212159

[13] Holt JA Luo G Billin AN Bisi J McNeill YY Kozarsky KF Donahee M Wang DY Mansfield TA Kliewer SA Goodwin B Jones SA Definition of a novel growth factor-dependent signal cascade for the suppression of bile acid biosynthesis. Genes Dev 17: 1581–1591, 2003 12815072 10.1101/gad.1083503 PMC196131

[14] Inagaki T Choi M Moschetta A Peng L Cummins CL McDonald JG Luo G Jones SA Goodwin B Richardson JA Gerard RD Repa JJ Mangelsdorf DJ Kliewer SA Fibroblast growth factor 15 functions as an enterohepatic signal to regulate bile acid homeostasis. Cell Metab 2: 217–225, 2005 16213224 10.1016/j.cmet.2005.09.001

[15] Inagaki T Moschetta A Lee YK Peng L Zhao G Downes M Yu RT Shelton JM Richardson JA Repa JJ Mangelsdorf DJ Kliewer SA Regulation of antibacterial defense in the small intestine by the nuclear bile acid receptor. Proc Natl Acad Sci USA 103: 3920–3925, 2006 16473946 10.1073/pnas.0509592103 PMC1450165

[16] Jelinek DF Andersson S Slaughter CA Russell DW Cloning and regulation of cholesterol 7 alpha-hydroxylase, the rate-limiting enzyme in bile acid biosynthesis. J Biol Chem 265: 8190–8197, 1990 2335522 PMC4451855

[17] Jung D Fantin AC Scheurer U Fried M Kullak-Ublick GA Human ileal bile acid transporter gene ASBT (SLC10A2) is transactivated by the glucocorticoid receptor. Gut 53: 78–84, 2004 14684580 10.1136/gut.53.1.78 PMC1773940

[18] Keitel V Haussinger D Perspective: TGR5 (Gpbar-1) in liver physiology and disease. Clin Res Hepatol Gastroenterol 36: 412–419, 2012 22521118 10.1016/j.clinre.2012.03.008

[19] Lenicek M Duricova D Komarek V Gabrysova B Lukas M Smerhovsky Z Vitek L Bile acid malabsorption in inflammatory bowel disease: Assessment by serum markers. Inflamm Bowel Dis 17: 1322–1327, 2011 21058331 10.1002/ibd.21502

[20] Lenicek M Duricova D Komarek V Gabrysova B Lukas M Smerhovsky Z Vitek L Bile acid malabsorption in inflammatory bowel disease: Assessment by serum markers. Inflamm Bowel Dis 17: 1322–1327, 2010 21058331 10.1002/ibd.21502

[21] Li-Hawkins J Gafvels M Olin M Lund EG Andersson U Schuster G Bjorkhem I Russell DW Eggertsen G Cholic acid mediates negative feedback regulation of bile acid synthesis in mice. J Clin Invest 110: 1191–1200, 2002 12393855 10.1172/JCI16309 PMC150802

[22] Li T Holmstrom SR Kir S Umetani M Schmidt DR Kliewer SA Mangelsdorf DJ The G protein-coupled bile acid receptor, TGR5, stimulates gallbladder filling. Mol Endocrinol 25: 1066–1071, 2011 21454404 10.1210/me.2010-0460 PMC3100601

[23] Lindor KD Farnesoid X receptor agonists for primary biliary cirrhosis. Curr Opin Gastroenterol 27: 285–288, 2011 21297469 10.1097/MOG.0b013e32834452c8

[24] Lundasen T Galman C Angelin B Rudling M Circulating intestinal fibroblast growth factor 19 has a pronounced diurnal variation and modulates hepatic bile acid synthesis in man. J Intern Med 260: 530–536, 2006 17116003 10.1111/j.1365-2796.2006.01731.x

[25] Makishima M Okamoto AY Repa JJ Tu H Learned RM Luk A Hull MV Lustig KD Mangelsdorf DJ Shan B Identification of a nuclear receptor for bile acids. Science 284: 1362–1365, 1999 10334992 10.1126/science.284.5418.1362

[26] Maruyama T Miyamoto Y Nakamura T Tamai Y Okada H Sugiyama E Itadani H Tanaka K Identification of membrane-type receptor for bile acids (M-BAR). Biochem Biophys Res Commun 298: 714–719, 2002 12419312 10.1016/s0006-291x(02)02550-0

[27] Mi LZ Devarakonda S Harp JM Han Q Pellicciari R Willson TM Khorasanizadeh S Rastinejad F Structural basis for bile acid binding and activation of the nuclear receptor FXR. Mol Cell 11: 1093–1100, 2003 12718893 10.1016/s1097-2765(03)00112-6

[28] Miyata M Hata T Yamakawa H Kagawa T Yoshinari K Yamazoe Y Involvement of multiple elements in FXR-mediated transcriptional activation of FGF19. J Steroid Biochem Mol Biol 132: 41–47, 2012 22561792 10.1016/j.jsbmb.2012.04.008

[29] Morelli A Comeglio P Filippi S Sarchielli E Cellai I Vignozzi L Yehiely-Cohen R Maneschi E Gacci M Carini M Adorini L Vannelli GB Maggi M Testosterone and farnesoid X receptor agonist INT-747 counteract high fat diet-induced bladder alterations in a rabbit model of metabolic syndrome. J Steroid Biochem Mol Biol 132: 80–92, 2012 22406511 10.1016/j.jsbmb.2012.02.007

[30] Neimark E Chen F Li X Shneider BL Bile acid-induced negative feedback regulation of the human ileal bile acid transporter. Hepatology 40: 149–156, 2004 15239098 10.1002/hep.20295

[31] Parks DJ Blanchard SG Bledsoe RK Chandra G Consler TG Kliewer SA Stimmel JB Willson TM Zavacki AM Moore DD Lehmann JM Bile acids: natural ligands for an orphan nuclear receptor. Science 284: 1365–1368, 1999 10334993 10.1126/science.284.5418.1365

[32] Pellicciari R Fiorucci S Camaioni E Clerici C Costantino G Maloney PR Morelli A Parks DJ Willson TM 6alpha-ethyl-chenodeoxycholic acid (6-ECDCA), a potent and selective FXR agonist endowed with anticholestatic activity. J Med Chem 45: 3569–3572, 2002 12166927 10.1021/jm025529g

[33] Richter HG Benson GM Blum D Chaput E Feng S Gardes C Grether U Hartman P Kuhn B Martin RE Plancher JM Rudolph MG Schuler F Taylor S Bleicher KH Discovery of novel and orally active FXR agonists for the potential treatment of dyslipidemia & diabetes. Bioorg Med Chem Lett 21: 191–194, 2011 21134747 10.1016/j.bmcl.2010.11.039

[34] Sinha J Chen F Miloh T Burns RC Yu Z Shneider BL beta-Klotho and FGF-15/19 inhibit the apical sodium-dependent bile acid transporter in enterocytes and cholangiocytes. Am J Physiol Gastrointest Liver Physiol 295: G996–G1003, 2008 18772362 10.1152/ajpgi.90343.2008 PMC2584833

[35] Song KH Li T Owsley E Strom S Chiang JY Bile acids activate fibroblast growth factor 19 signaling in human hepatocytes to inhibit cholesterol 7alpha-hydroxylase gene expression. Hepatology 49: 297–305, 2009 19085950 10.1002/hep.22627 PMC2614454

[36] van Tilburg AJ de Rooij FW van den Berg JW van Blankenstein M Primary bile acid diarrhoea without an ileal carrier defect: quantification of active bile acid transport across the ileal brush border membrane. Gut 32: 500–503, 1991 2040472 10.1136/gut.32.5.500 PMC1378925

[37] van Tilburg AJ de Rooij FW van den Berg JW van Blankenstein M Primary bile acid malabsorption: a pathophysiologic and clinical entity? Scand J Gastroenterol Suppl 194: 66–70, 1992 1298051 10.3109/00365529209096030

[38] Walters JR Howard A Rumble HE Prathalingam SR Shaw-Smith CJ Legon S Differences in expression of homeobox transcription factors in proximal and distal human small intestine. Gastroenterology 113: 472–477, 1997 9247466 10.1053/gast.1997.v113.pm9247466

[39] Walters JR Tasleem AM Omer OS Brydon WG Dew T le Roux CW A new mechanism for bile acid diarrhea: defective feedback inhibition of bile acid biosynthesis. Clin Gastroenterol Hepatol 7: 1189–1194, 2009 19426836 10.1016/j.cgh.2009.04.024

[40] Wang H Chen J Hollister K Sowers LC Forman BM Endogenous bile acids are ligands for the nuclear receptor FXR/BAR. Mol Cell 3: 543–553, 1999 10360171 10.1016/s1097-2765(00)80348-2

[41] Wistuba W Gnewuch C Liebisch G Schmitz G Langmann T Lithocholic acid induction of the FGF19 promoter in intestinal cells is mediated by PXR. World J Gastroenterol 13: 4230–4235, 2007 17696253 10.3748/wjg.v13.i31.4230 PMC4250623

[42] Yu J Lo JL Huang L Zhao A Metzger E Adams A Meinke PT Wright SD Cui J Lithocholic acid decreases expression of bile salt export pump through farnesoid X receptor antagonist activity. J Biol Chem 277: 31441–31447, 2002 12052824 10.1074/jbc.M200474200

[43] Zweers SJ Booij KA Komuta M Roskams T Gouma DJ Jansen PL Schaap FG The human gallbladder secretes fibroblast growth factor 19 into bile: towards defining the role of fibroblast growth factor 19 in the enterobiliary tract. Hepatology 55: 575–583, 2012 21953282 10.1002/hep.24702

